# NNMT and the methylation sink: integrating metabolism, epigenetics and immunity in cancer

**DOI:** 10.1186/s12916-026-04986-7

**Published:** 2026-06-12

**Authors:** Lvyuan Li, Yichun Ma, Yani Pan, Bowen Li, Chen Chen, Qiang Wang, Yao Fu, Xiangshan Fan, Shouyu Wang, Zhangding Wang

**Affiliations:** 1 Department of Pathology, The First Affiliated Hospital of Anhui Medical University, Hefei, 230000 Anhui China; 2 Department of Pathology, Affiliated Hospital of Medical School, Nanjing Drum Tower Hospital, Nanjing University, Nanjing, 210000 Jiangsu China; 3 Department of Gastroenterology, Affiliated Hospital of Medical School, Nanjing Drum Tower Hospital, Nanjing University, Nanjing, 210000 Jiangsu China; 4 Department of Hepatobiliary Surgery, MOE Innovation Center for Basic Research in Tumor Immunotherapy, Anhui Province Key Laboratory of Tumor Immune Microenvironment and Immunotherapy, The First Affiliated Hospital of Anhui Medical University, Hefei, 230000 Anhui China; 5 Department of Pathology, School of Basic Medical Sciences, Anhui Medical University, Hefei, 230032 Anhui China

**Keywords:** NNMT, MNAM, Methylation sink, Cancer metabolism, Epigenetics, Tumor microenvironment, NNMT inhibitors.

## Abstract

Nicotinamide N-methyltransferase (NNMT) is a methyltransferase that uses S-adenosyl-L-methionine (SAM, cofactor) to catalyze the N-methylation of nicotinamide (NAM, substrate), yielding 1-methylnicotinamide (MNAM) and S-adenosyl-homocysteine (SAH). By consuming SAM and generating SAH, NNMT establishes a cellular “methylation sink” that couples metabolic reprogramming to epigenetic remodeling across DNA, RNA and proteins. Accumulating evidence shows that NNMT is upregulated in multiple malignancies, across both cancer cells and stromal lineages such as cancer-associated fibroblasts and pericytes. Its activity correlates with key hallmarks of cancer progression, including tumor growth, metastasis potential, metabolic rewiring, immune evasion, angiogenesis, maintenance of stem-like states, and resistance to therapy (including chemotherapy, targeted agents, and radiotherapy). These properties nominate NNMT as a candidate biomarker for diagnosis and stratification and as a tractable therapeutic node. We synthesize current knowledge of NNMT-driven cellular and microenvironmental mechanisms in tumorigenesis and progression, and summarize emerging therapeutic strategies, which include competitive inhibitors targeting the substrate or cofactor binding sites, microenvironment-activated prodrugs, and rational combinations with immune checkpoint blockade and targeted therapy to provide a conceptual and translational framework for developing NNMT-directed interventions.

## Background

Nicotinamide N-methyltransferase (NNMT) is a one-carbon methyltransferase that transfers a methyl group from S-adenosyl-L-methionine (SAM) to nicotinamide (NAM; a precursor in the NAD⁺ salvage pathway), producing S-adenosyl-L-homocysteine (SAH) and 1-methylnicotinamide (MNAM; also reported as MNA) [1]. By consuming SAM and generating SAH, NNMT effectively depresses the cellular methyl-donor pool and imposes product inhibition on methyltransferases, thereby altering methylation states across DNA, RNA, proteins and selected secondary metabolites and, in turn, reshaping energy metabolism and epigenetic regulation.

Physiologically, NNMT exhibits a tissue-restricted expression pattern, with highest expression in the liver, lower levels in heart, placenta, lung, skeletal muscle and kidney, and little to no expression in the brain or pancreas [2]. This distribution suggests that baseline NNMT expression is not uniformly high across normal epithelial tissues, an important consideration when interpreting its expression changes in epithelial malignancies. NNMT was first partially purified from rat liver by Cantoni, laying the groundwork for subsequent biochemical characterization [3]. Early work primarily framed NNMT primarily as an enzyme in NAM catabolism and xenobiotic detoxification [2, 4–7]. With expanded investigation, however, NNMT has emerged as a broader regulator of metabolic and pathological processes [8–10]. In oncology, NNMT has emerged as a metabolism-linked factor whose expression and function vary according to tumor type, cellular lineage, and microenvironmental context [11]. Rather than showing uniform upregulation across all cancers, NNMT appears to be dysregulated in a context-dependent manner, with increased expression reported in several malignancies and often linked to metastasis, therapy resistance, or unfavorable clinical outcome [12–15].

Functionally, NNMT acts as a regulatory nexus between cellular metabolism and epigenetic control. Tumor cells and stromal cells (for example, cancer-associated fibroblasts) that upregulate NNMT frequently exhibit epigenetic alterations, including reduced methylation of histones and other cancer-associated proteins, as well as hypomethylation at the DNA and RNA levels, together with enhanced expression of oncogenic products [16], changes that collectively promote malignant progression [13, 14]. Importantly, the NNMT product MNAM is not an inert end-metabolite [17]; within the tumor microenvironment it can dampen T-cell function, thereby facilitating immune evasion and tumor growth [18]. Thus, dysregulated NNMT contributes to tumorigenesis and progression through coordinated epigenetic remodeling and metabolite-driven signaling.

Given that cancer remains a leading cause of mortality worldwide [19], early detection, accurate prognostic assessment, and the development of novel therapeutic interventions are crucial for achieving successful cancer treatment [20]. Preclinical studies in vitro and in vivo indicate that pharmacological inhibition of NNMT curtails cancer-cell proliferation and reduces tumorigenicity in murine models [21], underscoring NNMT as a tractable therapeutic target.

In this Review, we synthesize high-quality evidence on NNMT in malignant progression, detailing how NNMT influences proliferation, metastasis, metabolic reprogramming, immune escape, angiogenesis, therapy resistance, stemness and radioresistance, and further discuss the regulatory networks governing NNMT expression. We also collate current NNMT-directed therapeutic strategies to inform drug development and rational combinations, providing a conceptual framework for clinical translation.

### NNMT-mediated metabolic regulation

**Fig. 1:**
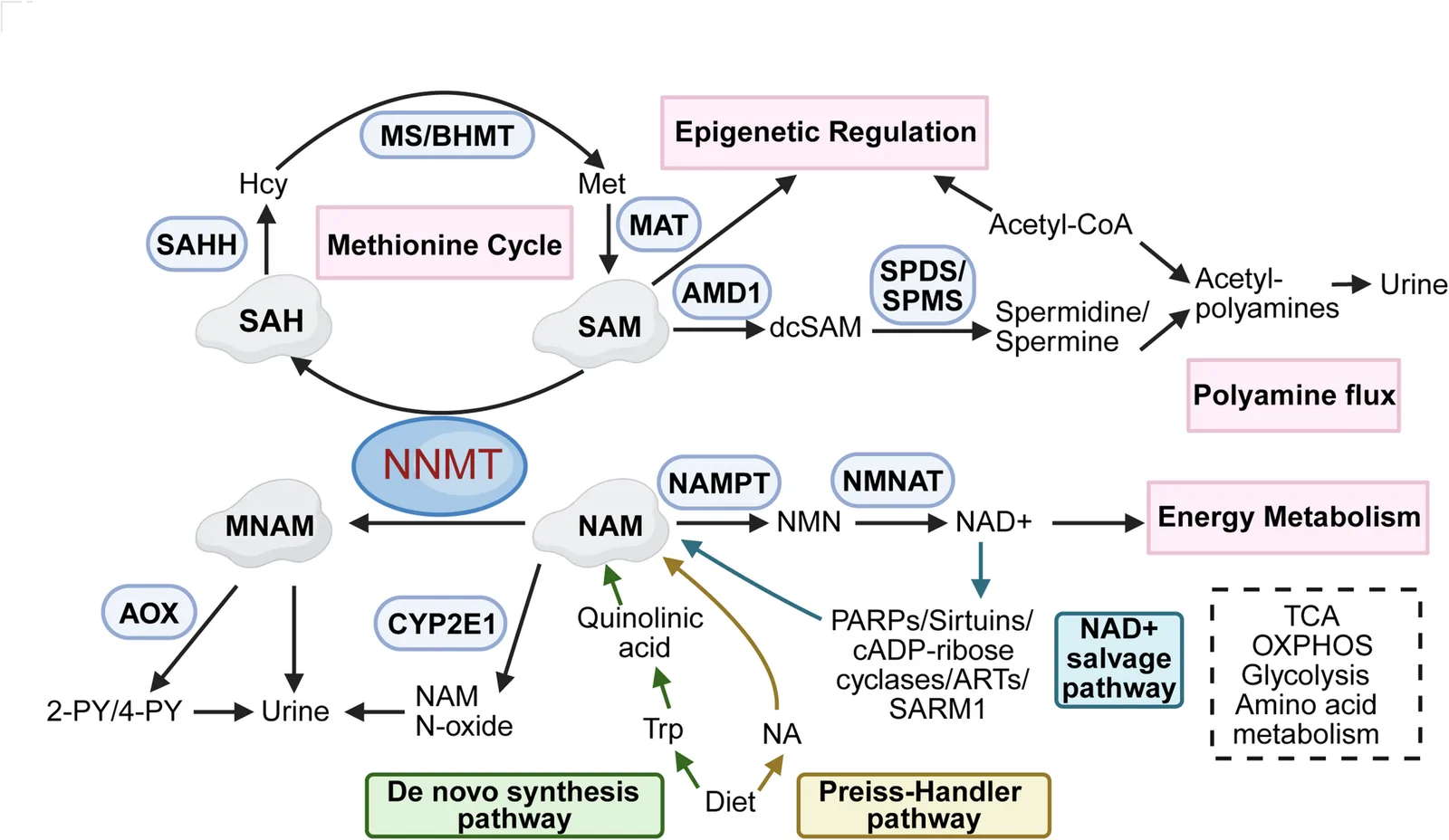
Metabolic processes mediated by NNMT. The schematic illustration shows the role of NNMT in the methionine cycle, epigenetic regulation, polyamine flux, and NAD^+^-related metabolic signaling pathways. Trp, Tryptophan; Met, Methionine; SAM, S-adenosyl-L-methionine; NAM, Nicotinamide; MNAM, 1-methylnicotinamide; SAH, S-adenosyl-homocysteine; AOX, Aldehyde oxidase; 2-PY, N^1^-methyl-2-pyridone-5-carboxamide; 4-PY, N^1^-methyl-4-pyridone-3-carboxamide; SAHH, SAH hydrolase; Hcy, Homocysteine; MS, Methionine synthase; BHMT, Betaine–homocysteine S-methyltransferase; MAT, Methionine adenosyltransferase; AMD1, Adenosylmethionine decarboxylase 1; dcSAM, Decarboxylated SAM; SPDS, Spermidine synthase; SPMS, Spermine synthase; SSAT, Spermidine–spermine N^1^-acetyltransferase; NA, Nicotinic acid; PARPs, Poly(ADP-ribose) polymerases; ARTs, ADP-ribosyl transferases; NMN, Nicotinamide mononucleotide; NAMPT, Nicotinamide phosphoribosyltransferase; NMNAT, NMN adenylyltransferase; CYP2E1, Cytochrome P450 2E1; TCA, Tricarboxylic-acid; OXPHOS, Oxidative phosphorylation

NNMT transfers the activated methyl group from the universal methyl donor SAM to NAM, yielding the stable metabolite MNAM together with SAH [22–24]. NNMT exhibits the canonical N-methyltransferase fold, with its SAM-binding domain and substrate-binding domain positioned adjacent to each other in space but separated by an α-helix. Together, they form a deeply buried hydrophobic pocket at the protein core, the access to which is gated by an N‑terminal “cap” structure [25]. MNAM can be excreted directly in urine, or oxidized by aldehyde oxidase (AOX) to N^1^-methyl-2-pyridone-5-carboxamide (2-PY) and N^1^-methyl-4-pyridone-3-carboxamide (4-PY), which are subsequently cleared renally [4]. Among urinary products, MNAM and 2-PY generally predominate, accounting for approximately 20–35% and 45–60% of NNMT-derived metabolites, respectively [26]. Beyond NAM clearance, NNMT activity interfaces with the NAD⁺-linked metabolic signaling, polyamine flux, and methionine cycle, thereby influencing epigenetic modifications (Fig. 1).

NAM is a metabolite of NAD⁺ and a key precursor for its salvage synthesis. It is primarily derived from three pathways, with the salvage pathway contributing to over 85% of cellular NAD⁺ production [27, 28]. This pathway utilizes NAD⁺ as a substrate to generate NAM through the catabolic activities of five classes of enzymes [29–31]. Subsequently, NAM is reconverted to NAD⁺ via sequential catalysis by NAMPT and NMNAT [32]. NAD⁺ levels are often inversely correlated with Hcy levels [33] and, serving as a central coenzyme, NAD⁺ participates in energy metabolism processes including the TCA cycle and OXPHOS [34]. Furthermore, NAM influences cell viability and metabolic function either directly or by modulating the balance of NAD⁺ and tryptophan metabolism [35]. Excess NAM can be oxidized by CYP2E1 to NAM N-oxide and excreted in urine [5].

Polyamine metabolism lies downstream of several oncogenic pathways and are frequently dysregulated in cancer, with established roles in tumor progression [36]. The SAM utilized by NNMT can serve as a substrate for the synthesis of polyamines. High NNMT expression creates competition for SAM, potentially limiting its availability for polyamine synthesis. Furthermore, polyamine clearance requires acetylation, a process that consumes acetyl-CoA, which is also a key substrate for epigenetic modifications. This conversion to acetyl-polyamines is a prerequisite for their urinary excretion [23]. Therefore, NNMT may indirectly influence polyamine flux and the metabolic partitioning of acetyl-CoA through these intricate interactions.

In the methionine cycle, SAH generated through a reaction catalyzed by NNMT can be hydrolyzed to form Hcy. Subsequently, Hcy is converted to methionine under the catalysis of MS or BHMT [37, 38], which is then converted into SAM by MAT, thereby completing the methionine cycle. Through this cycle, NNMT effectively compresses methyl-donor potential (often reflected by a lowered SAM/SAH ratio) and can indirectly modulate the activity of multiple DNA, RNA and protein methyltransferases.

Collectively, by coupling SAM consumption and SAH generation to NAM consumption and MNAM/2-PY/4-PY production, NNMT sits at the crossroads of methyl-group economy, polyamine biogenesis and NAD⁺ salvage. These connections position NNMT as a key rheostat of metabolic and epigenetic homeostasis, with broad implications for cancer-cell state and tumor microenvironmental signaling.

### Context-dependent roles of NNMT in tumorigenesis and progression

As the catalytic sink for the universal methyl donor SAM, NNMT converts SAM and NAM into SAH and MNAM. By depleting SAM and elevating SAH, NNMT depresses cellular methyl-transfer potential and thereby attenuates methylation across histones and non-histone proteins as well as DNA and RNA. In parallel, NNMT-driven metabolic rewiring can secondarily reprogrammed gene expression. Owing to inter- and intra-tumoral heterogeneity, these mechanisms manifest with context-dependent phenotypes across tumor types and cellular compartments.

### NNMT in tumor stromal cells

**Fig. 2:**
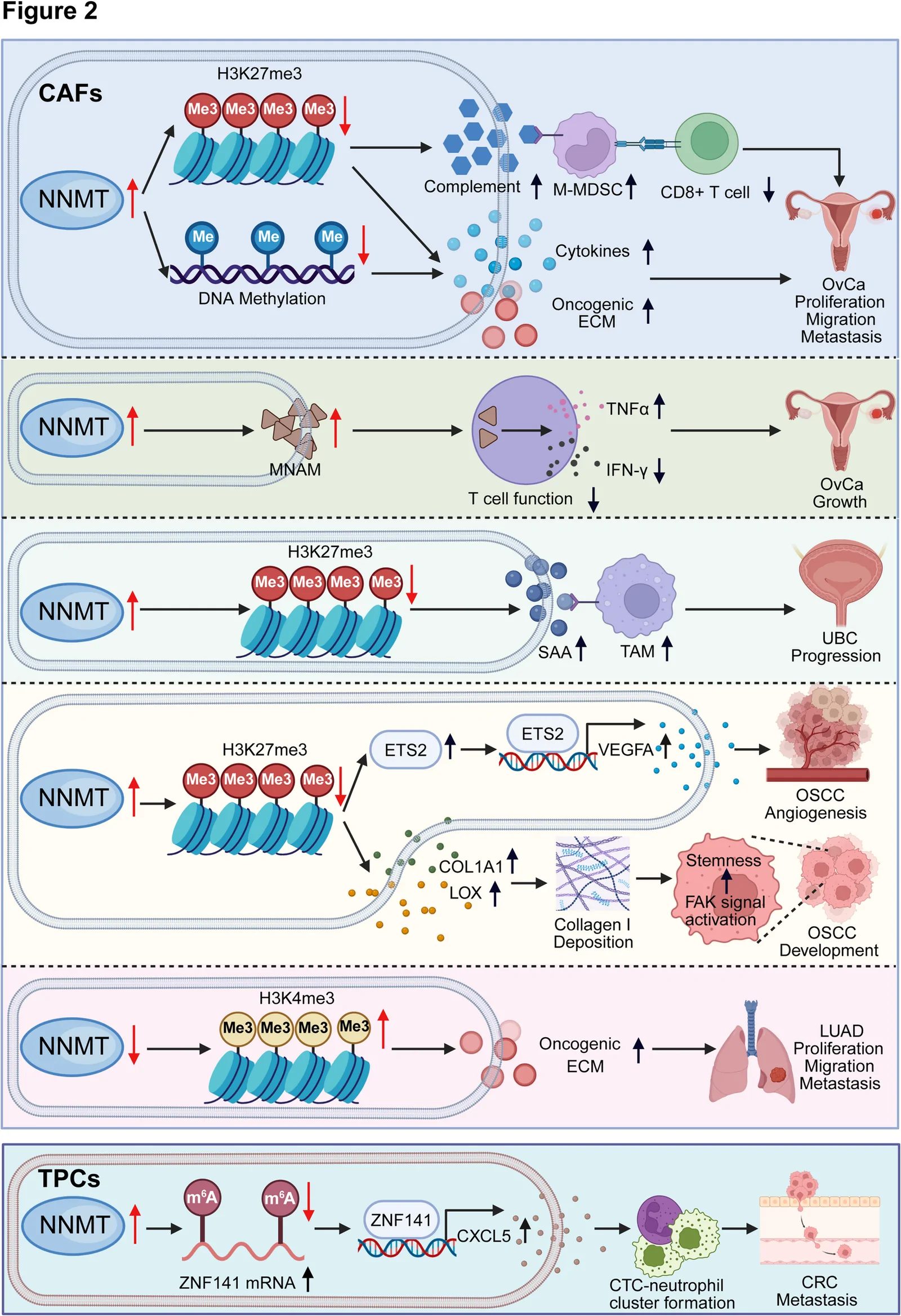
Mechanisms by which altered NNMT expression in stromal cells promotes malignant tumor progression. Altered NNMT expression in cancer-associated fibroblasts promotes tumor malignancy (upper panel). Upregulated NNMT expression in pericytes promotes the malignant progression of colorectal cancer (lower panel). CAFs, Cancer-associated fibroblasts; M-MDSC, Monocytic myeloid-derived suppressor cell; OvCa, Ovarian cancer; TAM, Tumor-associated macrophage; UBC, Urothelial bladder cancer; OSCC, Oral squamous cell carcinoma; ECM, Extracellular matrix; LUAD, Lung adenocarcinoma; CTC, circulating tumor cell; CRC, Colorectal cancer; SAA, Serum amyloid A; FAK, Focal-adhesion kinase

Stromal cells are pivotal architects of the tumor microenvironment (TME) and critically shape cancer progression [39]. Dissecting stromal pro-tumorigenic mechanisms is therefore essential for biomarker discovery and therapeutic targeting. Among these mechanisms, NNMT has emerged as a key stromal regulator (Fig. 2).

As the most active stromal lineage, cancer-associated fibroblasts (CAFs) remodel extracellular matrix (ECM) and secrete cytokines and growth factors that directly promote tumor progression [40]. NNMT is frequently upregulated in CAFs, where SAM depletion and SAH accumulation lower DNA and histone methylation, most notably histone H3 lysine 27 trimethylation (H3K27me3), thereby reprogramming gene expression towards pro-tumor ECM and cytokine signatures and accelerating ovarian-cancer growth and metastasis [41]. Extending this paradigm, NNMT-induced loss of H3K27me3 in CAFs activates complement components, recruits monocytes and drives an immunosuppressive monocytic myeloid-derived suppressor cell (M-MDSC) programing in the TME, collectively restraining CD8⁺ T-cell function [42]. NNMT-mediated metabolites also operate non-cell-autonomously, CAF-derived MNAM can be exported into the TME and taken up by T cells, where it elevates TNF-α while reducing IFN-γ, dampening T-cell effector function and facilitating ovarian-cancer growth [18].

In urothelial bladder cancer, high NNMT in CAFs rewires histone methylation (decreased H3K27me3) to upregulate serum amyloid A (SAA), which recruits and polarizes macrophages toward tumor-associated phenotypes, fueling progression and conferring resistance to PD-1/PD-L1 immune checkpoint blockade [43]. In oral squamous cell carcinoma (OSCC), NNMT-high CAFs suppress H3K27me3 to induce ETS2, which in turn upregulates VEGFA, promoting angiogenesis and tumor expansion [44]. In parallel, NNMT-dependent H3K27me3 loss enhances COL1A1 and LOX transcription, triggering LOX-mediated type-I collagen deposition, activation of focal-adhesion kinase (FAK) signaling in cancer cells and OSCC progression [45]. Given that NNMT expression levels are intrinsically heterogeneous across different tumor types, and that it regulates histone methylation broadly and non-specifically, the downstream phenotypes it modulates may therefore exhibit high heterogeneity in various cells and cancers. In lung adenocarcinoma, downregulation of NNMT in CAFs correlates with increased histone H3 lysine 4 trimethylation (H3K4me3) and induction of ECM-remodeling genes (collagens, integrins, laminins and matrix metalloproteinases), changes associated with heightened tumor cell invasion and metastasis [46]. Together, these observations position NNMT as a rheostat of CAF epigenomes that tunes pro-angiogenic, immunomodulatory and matrix-remodeling programming in a tumor-type-dependent fashion.

Pericytes are essential for vascular development and homeostasis [47], and their tumor counterparts (tumor pericytes, TPCs) contribute to growth, angiogenesis and metastatic dissemination [48, 49]. In colorectal cancer (CRC), pericyte NNMT reduces m^6^A modification on ZNF141 transcripts, thereby limiting YTHDF2-mediated mRNA decay and increasing ZNF141 protein; ZNF141 then elevates CXCL5, promoting the formation of circulating tumor cell (CTC)–neutrophil clusters and driving liver metastasis [50]. These data highlight an NNMT-centered stromal axis that couples epigenetic control (histone and RNA methylation) to immuno-vascular crosstalk and metastatic seeding. Thus, elucidating lineage-specific NNMT functions in CAFs and pericytes will refine therapeutic strategies that target stromal ecosystems and may inform combinations with immune checkpoint blockade and anti-angiogenic or anti-metastatic interventions.

### NNMT in cancer cells

Epigenetic dysregulation and metabolic reprogramming are two core engines of malignant transformation and progression that cooperatively enforce aggressive cancer phenotypes [51]. In cancer cells, NNMT overexpression accelerates consumption of the methyl donor SAM, thereby diminishing cellular methyl-transfer potential. This methyl-group drain not only perturbs metabolic homeostasis but also remodels epigenetic marks to rewire gene expression, collectively driving tumorigenesis and disease progression.

#### Protein-methylation–centered pathways

**Fig. 3:**
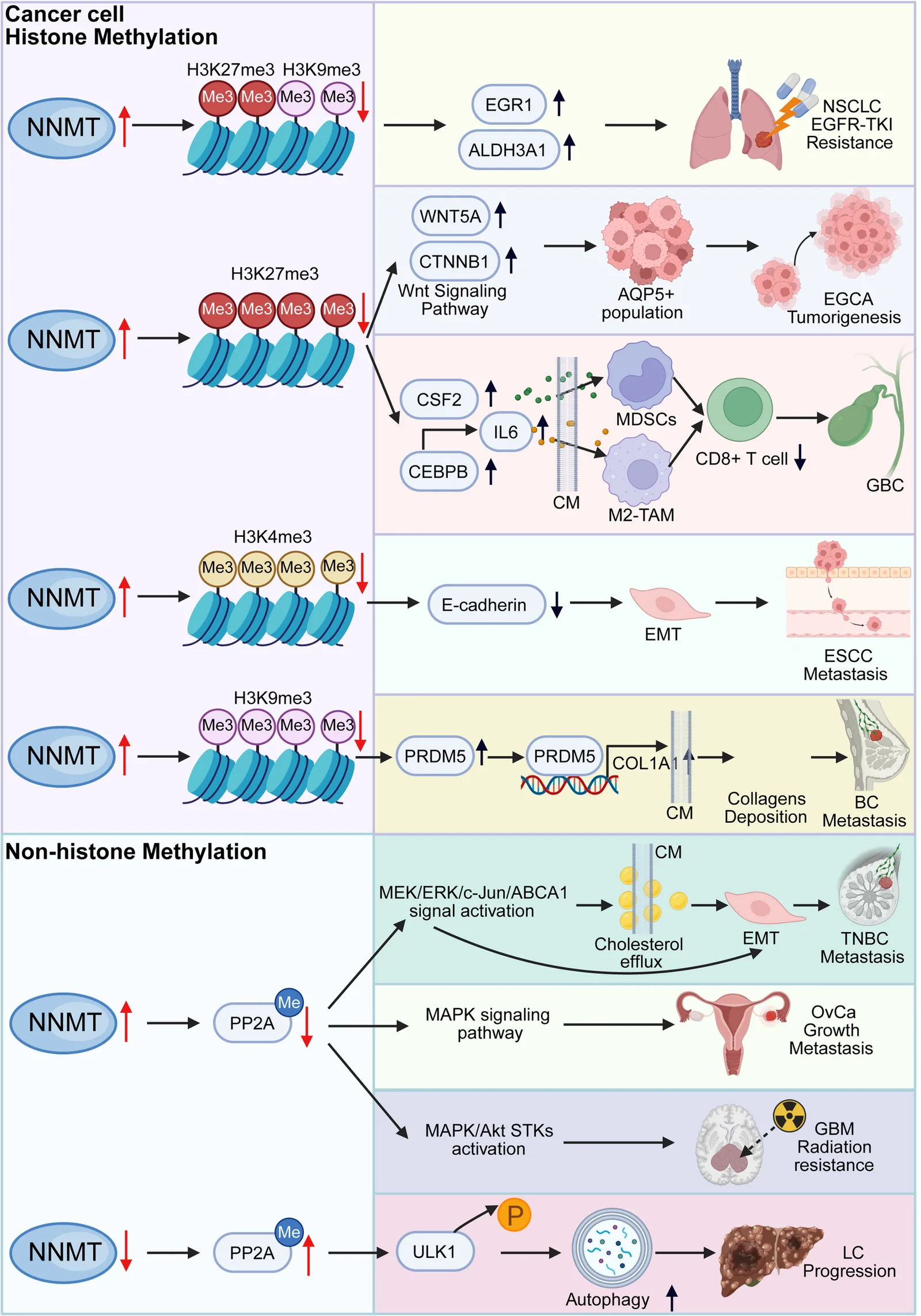
Mechanism by which NNMT promotes malignant cancer progression through regulating histone and non-histone methylation. NSCLC, Non-small-cell lung cancer; EGFR-TKI, Epidermal growth factor receptor tyrosine-kinase inhibitor; EGCA, Early gastric cardia adenocarcinoma; CM, Cell membrane; MDSCs, Myeloid-derived suppressor cell; M2-TAM, M2 tumor-associated macrophage; GBC, Gallbladder cancer; EMT, Epithelial–mesenchymal transition; ESCC, Esophageal squamous-cell carcinoma; BC, Breast cancer; TNBC, Triple-negative breast cancer; GBM, Glioblastoma; LC, Liver cancer; PP2A, Protein phosphatase 2 A; STKs, Serine/threonine kinases

By depleting SAM and elevating SAH, NNMT markedly reduces global methylation capacity, constraining both histone and non-histone methylation and, in turn, altering key signaling networks (Fig. 3). These changes underpin tumor-cell phenotypes linked to therapeutic resistance, stemness, immune evasion and metastatic potential.

NNMT lowers the levels of repressive histone marks and thereby activates oncogenic transcriptional circuits. In non-small-cell lung cancer (NSCLC), NNMT reduces histone H3 lysine 9 trimethylation (H3K9me3) and H3K27me3, epigenetically activating EGR1 and ALDH3A1 to promote resistance to epidermal growth factor receptor tyrosine-kinase inhibitors (EGFR-TKIs) [15]. In our prior work on AQP5⁺ cells, elevated NNMT reduced H3K27me3 and activated WNT signaling to maintain AQP5⁺ cancer stem-cell properties, fueling early gastric cardia adenocarcinoma (EGCA) progression [14]. In gallbladder cancer (GBC), NNMT decreases H3K27me3 at the promoters of IL6, CSF2 (encoding GM-CSF) and CEBPB (a key IL6 transcription factor), upregulating IL-6 and GM-CSF, skewing macrophages toward an M2 tumor-associated state and converting peripheral monocytes into myeloid-derived suppressor cells, thus establishing an immunosuppressive TME [52]. In esophageal squamous-cell carcinoma (ESCC), NNMT reduces H3K4me3 mark to suppress E-cadherin expression, thereby promoting epithelial-mesenchymal transition (EMT) and metastasis [53]. Likewise, in breast cancer, NNMT downregulates H3K9me3 and DNA methylation at the PRDM5 promoter to elevate PRDM5, which in turn induces collagen-gene transcription; together with NNMT-induced DNA hypomethylation at collagen-gene promoters, this drives collagen transcription and enhances pulmonary colonization [11]. These results indicate that NNMT does not regulate all histone methylation events, which may depend on the relative Km and Ki values of individual methyltransferases for SAM and SAH in different tissues [16].

Studies on non-histone protein methylation have revealed that NNMT modulates the methylation status of protein phosphatase 2 A (PP2A), with profound effects on its function and activity. NNMT lowers PP2A methylation and activity, unleashing MEK/ERK/c-JUN/ABCA1 signaling, increasing cholesterol efflux and membrane fluidity, and promoting EMT and metastatic dissemination in triple-negative breast cancer (TNBC) [13]. Concordant findings in ovarian cancer show that NNMT-mediated PP2A demethylation activates MEK/ERK to support growth and metastasis [54]. Similarly, in glioblastoma (GBM), reduced PP2A methylation aligns with activation of oncogenic serine/threonine kinases (STKs) and therapy resistance [55]. Of note, the relationship between NNMT and PP2A can be context-dependent in liver cancer, low NNMT correlates with increased PP2A methylation and phosphatase activity, promoting ULK1 dephosphorylation, activating autophagy and enabling survival under nutrient stress [56].

Collectively, these studies position NNMT as an upstream rheostat of epigenetic and signaling states in cancer cells. Through histone-mark erosion and non-histone targets, NNMT reprograms transcriptional and kinase networks that sustain EMT, stemness, immune suppression and resistance to targeted agents, providing valuable insights for developing NNMT-directed interventions to overcome context-specific vulnerabilities.

#### Nucleic-acid methylation pathways

**Fig. 4:**
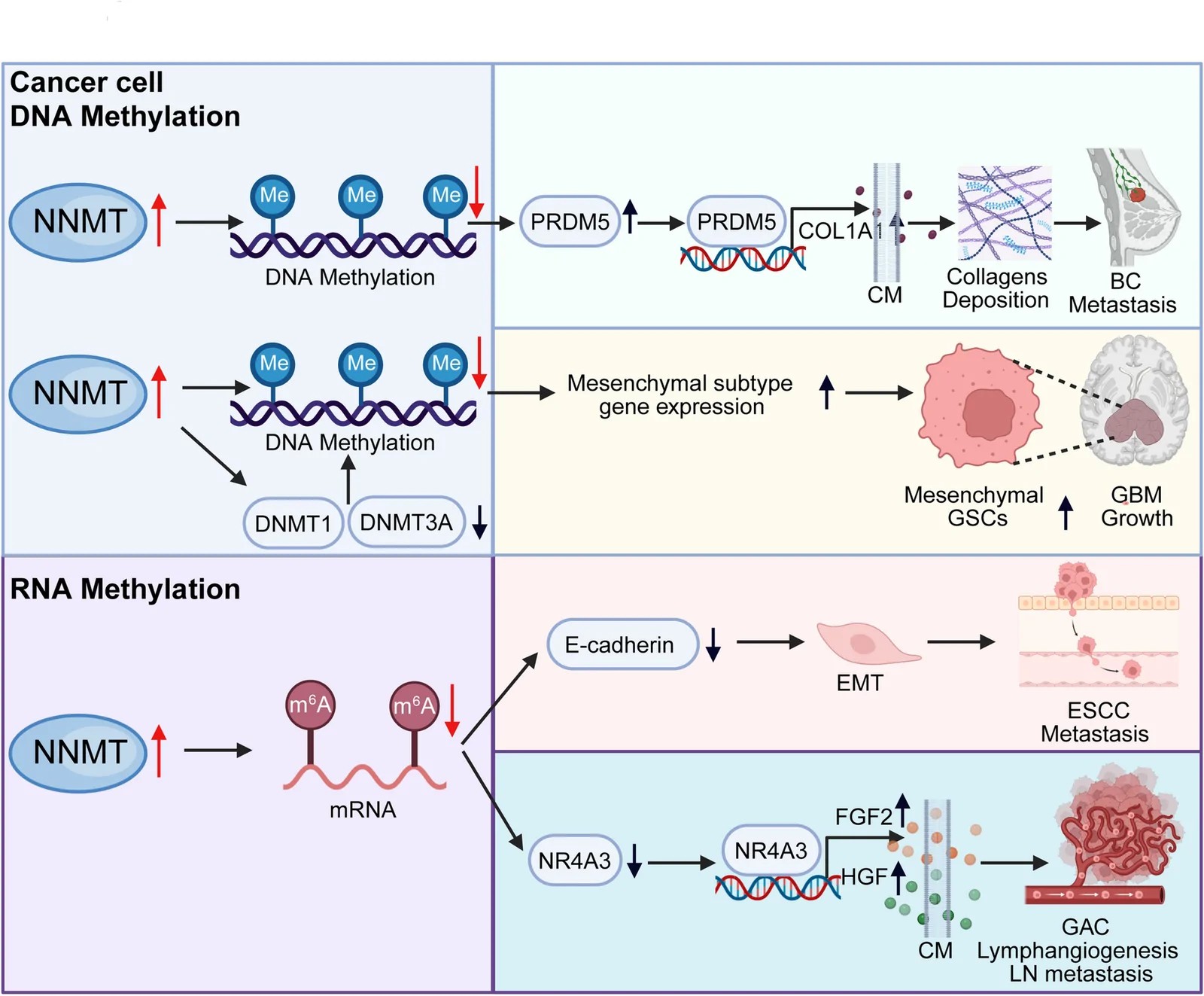
Mechanism by which NNMT promotes malignant cancer progression through regulating DNA and RNA methylation. CM, Cell membrane; BC, Breast cancer; GSCs, Glioblastoma stem cells; GBM, Glioblastoma; EMT, Epithelial-mesenchymal transition; ESCC, Esophageal squamous-cell carcinoma; GAC, gastric adenocarcinoma; LN, Lymph node; GSCs, Glioblastoma stem cells; m⁶A, N⁶-methyladenosine

By depleting SAM and elevating SAH, NNMT constrains the cellular methyl-transfer potential and imposes product inhibition on SAM-dependent methyltransferases. This not only dampens protein methylation but also broadly suppresses DNA and RNA methylation, thereby driving malignant progression through multiple mechanisms (Fig. 4).

DNA methylation is one of the core epigenetic modifications, and aberrant DNA methylation in cancer typically comprises global hypomethylation alongside focal promoter hypermethylation of tumor suppressor genes [57]. In breast cancer cells, NNMT lowers DNA methylation at the promoters of PRDM5 and collagen genes, enhancing their transcription and facilitating metastatic colonization [11]. In GBM, NNMT is overexpressed, reduces methyl-donor availability and downregulates DNMT1 and DNMT3A, collectively decreasing DNA methylation at mesenchymal-subtype gene loci. This epigenetic rewiring drives transcriptional conversion of proneural glioblastoma stem cells (GSCs) toward a mesenchymal program and accelerates tumor growth [58].

To date, more than one hundred chemical modifications have been catalogued on RNA [59]. Among them, N⁶-methyladenosine (m⁶A) installed within a conserved consensus [G/A/U][G > A]m⁶AC[U > A> C] [60, 61], is the most abundant internal mark on mRNA and is tightly linked to cancer progression with broad oncogenic implications [62, 63]. Beyond DNA methylation, NNMT modulates m⁶A-dependent post-transcriptional control. In ESCC, NNMT reduces m⁶A marks to suppress E-cadherin, thereby promoting EMT and metastasis [53]. In lymph node metastatic gastric adenocarcinoma (GAC), NNMT lowers m⁶A on NR4A3 mRNA, promoting its degradation; reduced NR4A3 occupancy at the FGF2 and HGF promoters lifts repression, increasing FGF2/HGF expression and driving lymphangiogenesis and lymphatic dissemination [64].

Through SAM depletion, NNMT coordinately suppresses DNA and mRNA methylation, leading to a concurrent reprogramming of both transcriptional and post-transcriptional landscapes. The resulting shift toward mesenchymal/EMT states, enhanced pro-metastatic matrix reprogramming and growth-factor signaling underscores NNMT as a central rheostat of nucleic-acid methylation in cancer cells.

#### Metabolic signaling pathways

**Fig. 5:**
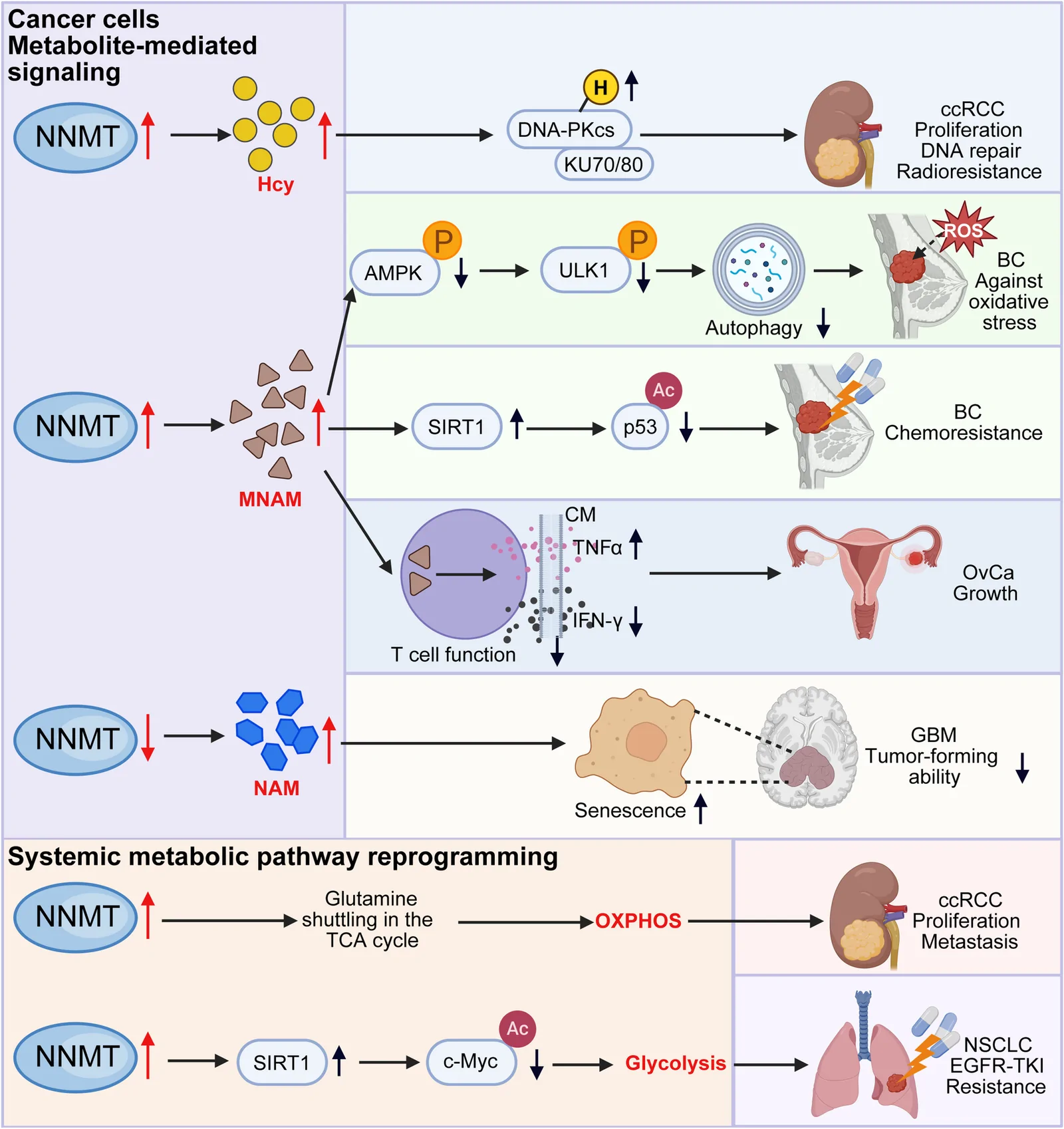
Mechanism by which NNMT promotes malignant cancer progression through metabolic regulation. ccRCC, Clear-cell renal cell carcinoma; BC, Breast cancer; CM, Cell membrane; OvCa, Ovarian cancer; GBM, Glioblastoma; NSCLC, Non-small-cell lung cancer; EGFR-TKI, Epidermal growth factor receptor tyrosine-kinase inhibitor; DNA-PKcs, DNA-dependent protein kinase catalytic subunit

As a key metabolic enzyme, NNMT drives tumorigenesis and progression through two mechanisms: direct metabolite-mediated signaling and systemic metabolic pathway reprogramming (Fig. 5). The primary product of NNMT, MNAM, inhibits the AMPK-ULK1 pathway, thereby reducing oxidative stress-induced autophagy in breast cancer cells and conferring a survival advantage to cancer cells under oxidative stress [65]. Concurrently, MNAM enhances breast cancer chemoresistance by stabilizing and enhancing the deacetylase activity of SIRT1 [66]. In addition to CAFs, MNAM is also secreted into the extracellular space by ovarian cancer cells, where it exerts similar functions [18]. Furthermore, NNMT induces homocysteinylation of DNA-dependent protein kinase catalytic subunits (DNA-PKcs) through the accumulation of the metabolic intermediate Hcy, thereby promoting cell proliferation, DNA repair, and resistance to radiotherapy in clear cell renal cell carcinoma (ccRCC) cells [67]. In a cell line derived from glioblastoma patients (GB-PDC), downregulation of NNMT leads to the accumulation of its substrate NAM, inducing cellular senescence and loss of tumorigenic properties [68].

On the other hand, NNMT promotes tumor progression by altering cellular metabolic states. Through the SIRT1/c-myc axis, NNMT promotes glycolysis and EGFR-TKI resistance in NSCLC cells [21]. Furthermore, NNMT supports oxidative phosphorylation in ccRCC by regulating the transport of glutamine within the tricarboxylic acid cycle, providing sufficient energy for cancer cell proliferation and promoting ccRCC growth and metastasis [69]. These studies indicate that NNMT plays a central role in tumor radiation resistance, autophagy regulation, chemotherapy resistance, immune evasion, cellular senescence, and metastasis through metabolic pathways, making it a key regulatory factor in tumor progression.

### Mechanisms regulating NNMT expression

**Fig. 6:**
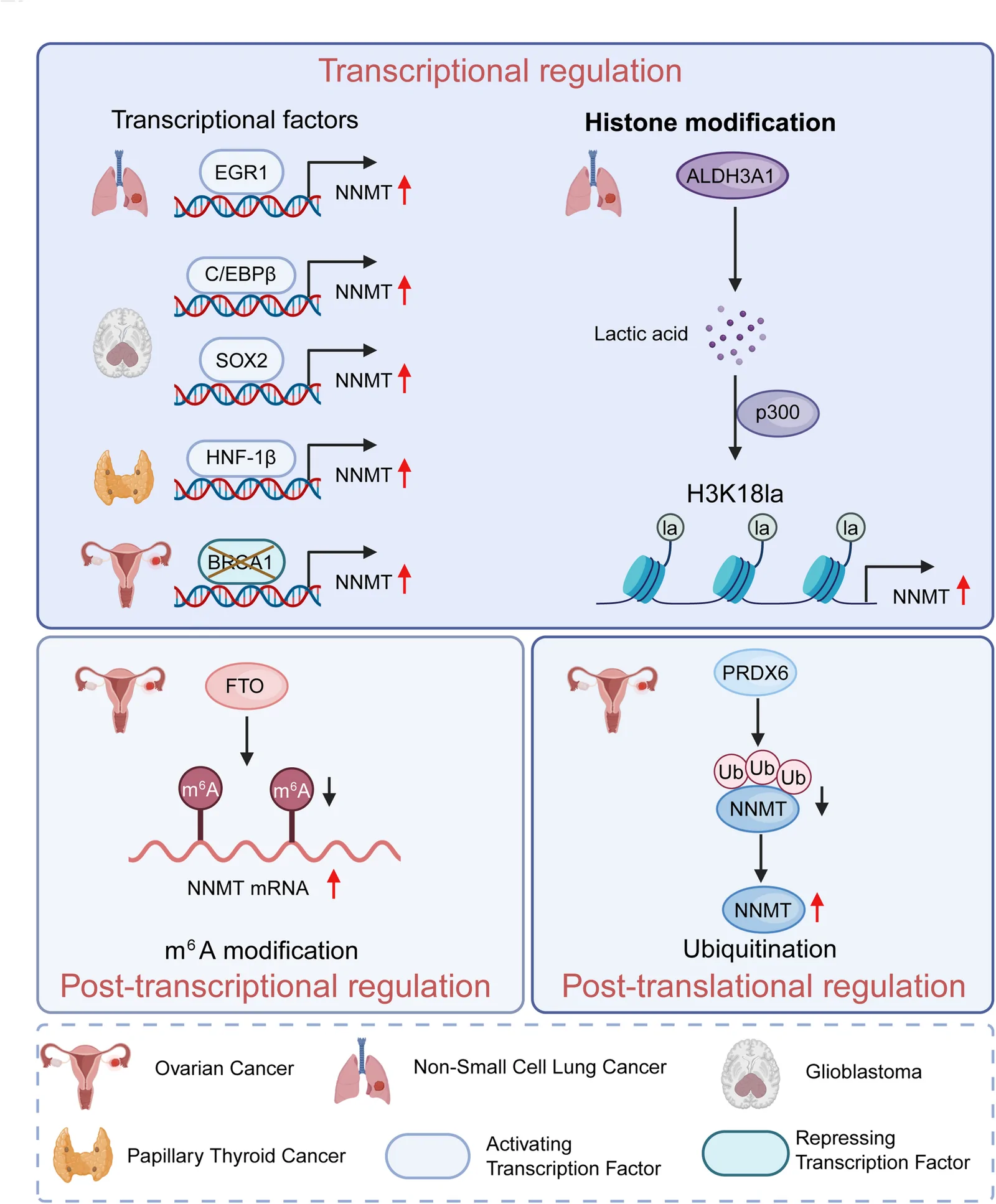
Mechanisms regulating NNMT expression. The schematic illustration depicts the upregulation of NNMT expression in cancer cells via transcriptional, post-transcriptional, and post-translational regulatory pathways

NNMT sits upstream of broad metabolic and epigenetic rewiring, and identifying the nodes that elevate NNMT provides actionable entry points to block tumor progression and overcome therapy resistance. Evidence indicates that NNMT expression is elevated in diverse tumor types through three major mechanisms: transcriptional activation (predominant), post-transcriptional modification, and proteostasis (inhibited degradation) (Fig. 6).

At the transcriptional level, several tumor-specific mechanisms promote NNMT upregulation. In NSCLC, EGR1 binds the NNMT promoter to directly activate transcription. In parallel, ALDH3A1 elevates lactate, which enhances p300-mediated histone H3 lysine 18 acetylation (H3K18la) at the NNMT promoter, further boosting NNMT expression [15]. In mesenchymal GBM, C/EBPβ transcriptionally induces key enzymes in the NAM metabolic pathway, including NNMT, reinforcing mesenchymal programming [58]. Another study on GBM showed that the SOX2 protein binds to the NNMT gene promoter, thereby upregulating NNMT expression [68]. In papillary thyroid cancer (PTC), HNF-1β functions as a transcriptional activator to upregulate NNMT [70]. In high-grade serous ovarian cancer, BRCA1 deficiency increases chromatin accessibility at the NNMT promoter, releasing repression and elevating NNMT expression [71]. At the post-transcriptional level, altered m^6^A modification contributes to NNMT upregulation. In platinum-sensitive ovarian cancer, high expression of the m6A demethylase FTO reduces methylation of NNMT transcripts, stabilizing its mRNA and enhancing expression, thereby modulating drug response [72]. At the protein level, stabilization mechanisms further sustain NNMT overexpression. For instance, PRDX6 interacts with NNMT in a non-enzymatic manner, preventing TRIM56-mediated ubiquitination and proteasomal degradation, thereby maintaining NNMT protein stability [54].

Together, these studies delineate a multilayer regulatory network that sustains NNMT overexpression through promoter activation (TF binding and histone acylation), mRNA modification (m⁶A dynamics) and protein-stability control (E3-ligase shielding). Therapeutically, targeting the upstream activators (EGR1, C/EBPβ, SOX2, HNF-1β), chromatin-modifying enzymes (p300/H3K18la), m⁶A machinery (FTO), or degradation pathways (TRIM56 axis) offers orthogonal strategies to down-tune NNMT and potentially reverse resistance phenotypes.

## NNMT as a therapeutic target

**Fig. 7:**
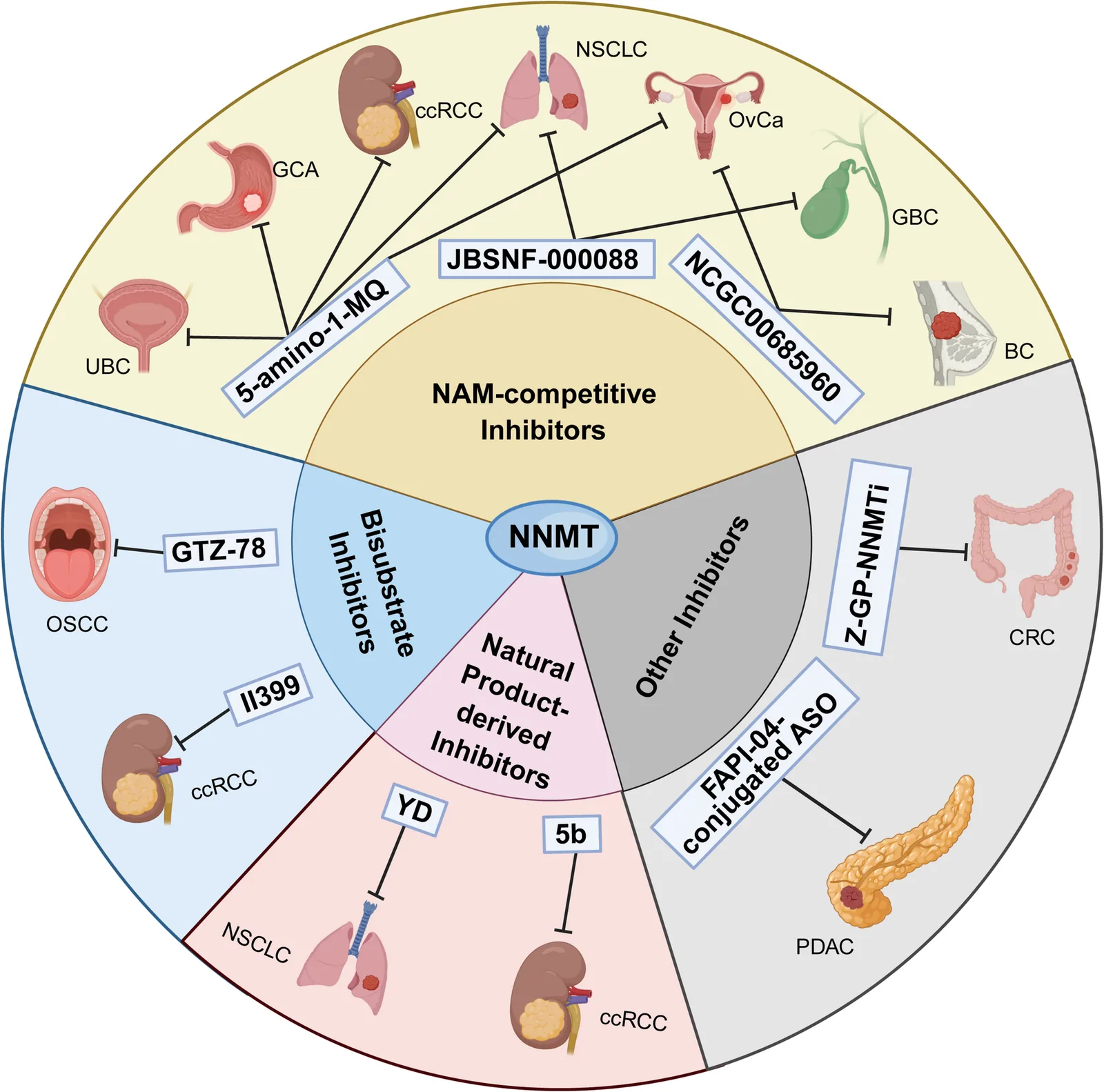
Inhibitors that can suppress NNMT expression in cancer cells. The schematic illustration shows four types of inhibitors that suppress NNMT expression in cancer cells: NAM-competitive inhibitors, bisubstrate inhibitors, natural product-derived inhibitors, and other inhibitors. UBC, Urothelial bladder cancer; GCA, gastric cardia adenocarcinoma; ccRCC, Clear-cell renal cell carcinoma; NSCLC, Non-small-cell lung cancer; OvCa, Ovarian cancer; GBC, Gallbladder cancer; BC, Breast cancer; CRC, Colorectal cancer; PDAC, Pancreatic ductal adenocarcinoma; OSCC, Oral squamous cell carcinoma; YD, Yuanhuadine; 5b, Alkaloid lycorine; ASO, Antisense oligonucleotide

Although no NNMT-directed therapy has yet entered routine clinical use, NNMT has gained momentum as a druggable metabolic-epigenetic node, with multiple chemical classes showing preclinical activity (Fig. 7).

Medicinal chemistry has largely pursued NNMT inhibitors (NNMTi) by mimicking NAM (substrate pocket) or SAM (cofactor pocket). Currently designed SAM-competitive inhibitors exhibit poor inhibitory activity [73, 74] and are insufficient to achieve highly selective inhibition of NNMT. Designing inhibitors that compete for binding with the NAM substrate is an effective strategy. The small-molecule NNMT inhibitor 5-amino-1-MQ exhibits high membrane permeability and can effectively inhibit NNMT activity at micromolar concentrations [75]. It has demonstrated efficacy in various mouse models, including ovarian cancer [41], gastric adenocarcinoma [14], bladder-urothelial-carcinoma [43], NSCLC [15], and ccRCC [69]. However, the primary challenge lies in the need to increase potency to the nanomolar range to meet clinical development requirements. JBSNF-000088 is a potent but slow-metabolizing inhibitor of NNMT [76]. JBSNF-000088 or gefitinib monotherapy mildly inhibited tumor growth, whereas the combination therapy significantly suppressed the in vitro and in vivo growth of EGFR-TKI-resistant NSCLC cells [21]. Treatment with JBSNF-000088 in GBC xenograft model overexpressing NNMT inhibited tumor progression and increased CD8⁺ T-cell infiltration [52]. Recently, Lengyel et al. used high-throughput screening to develop NCGC00685960, a potent, specific, and highly bioavailable NNMT inhibitor. It reduces tumor burden and metastasis in mouse models of ovarian and breast cancer. Moreover, it acts synergistically with anti-PD-1 or anti-CD47 to restore antitumor immunity [42].

Given the modest activity of compounds that target only the SAM or NAM pocket, “bisubstrate” designs that bridge both sites have been advanced to enhance potency and selectivity. Martin and colleagues structurally optimized a previously reported NNMT inhibitor by incorporating a naphthalene moiety, which markedly increased its activity [73]. The optimized compound, GTZ-78, inhibited proliferation of HSC-2 oral-cancer cells in a dose-dependent manner [77]. In parallel, Huang and colleagues developed a cell-active bisubstrate inhibitor, II399, by introducing an unconventional SAM analog. II399 displayed low-nanomolar binding affinity and significantly inhibited NNMT activity in the 769P human clear cell renal cell carcinoma cell line in vitro [78]. As bisubstrate inhibitors, the relatively high molecular weight of GTZ-78 and II399 may indeed pose challenges to cellular permeability, and their in vivo efficacy remains to be comprehensively validated.

In parallel with active-site inhibitors, several natural-product-derived agents reduce NNMT expression and thereby suppress tumor growth or drug resistance. The daphnane diterpenoid yuanhuadine (YD) downregulated NNMT and mitigated gefitinib resistance in NSCLC cells [79]. A methanesulfonamide analogue of the natural alkaloid lycorine (5b) lowered NNMT abundance and curtailed growth of ccRCC xenografts [80]. While mechanistically novel, the inherent polypharmacology of natural products complicates the attribution of their effects solely to NNMT inhibition, resulting in compromised clarity for their development as molecularly targeted agents.

Beyond direct inhibition, a major focus in NNMT-targeted research has been to achieve precise delivery or tumor-specific activation of therapeutics to maximize efficacy while minimizing systemic toxicity. A FAPI-04-conjugated antisense oligonucleotide (ASO), designed to home to tumor stroma, showed antitumor activity in a pancreatic-cancer model, illustrating a route to stromal-focused NNMT suppression while mitigating off-target effects typical of unconjugated ASOs [81]. In a syngeneic colorectal-cancer model, an enzyme-activated NNMT-inhibitor prodrug (Z-GP-NNMTi) curtailed MC38-LM3 liver metastases and reduced circulating CTC-neutrophil clusters [50], consistent with pericyte-selective activation and disruption of pro-metastatic niches. A shared translational challenge for both strategies is that their efficacy is critically dependent on the specificity, abundance, and homogeneity of distribution of the selected biomarker within tumor tissues, which may also carry a risk of off-target toxicity.

Collectively, substrate-competitive, bisubstrate and expression-modulating strategies, together with targeted delivery platforms, suggest that NNMT inhibition can restrain tumor growth, recondition the immune microenvironment, and overcome targeted-therapy resistance. These findings support the possibility that NNMT may serve as a potential therapeutic node and provide a preliminary rationale for exploring precision combination therapies in NNMT-high tumors. However, its clinical potential remains to be formally validated through early-phase clinical trials.

## Conclusions

Across multiple malignancies, including ovarian, bladder, oral squamous cell carcinoma, lung, colorectal, gastric, gallbladder, esophageal squamous cell carcinoma, breast, glioblastoma, clear-cell renal cell carcinoma, and hepatocellular carcinoma, NNMT plays a pivotal role in tumor growth, metastatic dissemination and therapeutic resistance. High, tumor-specific NNMT expression correlates with aggressive progression and poor clinical outcome [82], supporting its development both as a biomarker for diagnosis/stratification and as a tractable therapeutic target.

Therapeutically, the abundance of NNMT-high CAFs associates with diminished responses to immune checkpoint blockade (ICB)^[37, 38]^. This provides a rationale for combining NNMT inhibition with ICB to restore antitumor immunity, particularly in ICB-refractory settings. NNMT-derived metabolites can also act as indirect modulators of tumor progression and may suppress endogenous immune responses [18]. Further clarification of their functional roles could open new avenues for augmenting antitumor immunity. Building on the elevated expression of fibroblast activation protein-α in TPCs, enzyme-activated NNMT-inhibitor prodrugs (for example, Z-GP-NNMTi) enable pericyte-selective targeting, offering a precision route to remodel the TME [83]. More broadly, quantitative assessment of NNMT expression across tumor compartments should guide patient selection for NNMT-directed therapy, while the design of additional prodrug NNMT inhibitors and rational combinations promises to expand therapeutic options.

Companion diagnostics and pharmacodynamic monitoring will be key. The twelve-sulfonate pillar [6]arene (P6AS) functions as a high-affinity supramolecular biosensor for MNAM [84], enabling real-time readouts of NNMT activity in cancer. Such tools could support precision dosing, patient stratification and high-throughput discovery of NNMT inhibitors. Notably, although electron-deficient arene-alkene-linked bisubstrate mimics can inhibit NNMT with sub-nanomolar potency (IC₅₀ =3.7 nM) [85], their poor cell permeability limits cellular efficacy, underscoring a central medicinal-chemistry challenge for next-generation NNMT inhibitors. Although no NNMT-selective small molecules have yet entered clinical use, their development remains a key research priority, positioning NNMT as an emerging and highly attractive target in oncology.

Looking ahead, several scientific and translational priorities emerge. First, NNMT function appears context-dependent: for example, in lung adenocarcinoma, downregulation of NNMT in CAFs paradoxically enhances ECM-remodeling programming and promotes invasion/metastasis [46], raising the possibility of tumor-suppressive facets in specific niches. Systematic resolution of such dual or multiphasic roles, across tumor cells and stromal lineages, will be essential for safe, effective targeting. Second, methyltransferases may possess non-canonical functions [86]. Beyond its canonical enzymatic activity, whether NNMT participates in tumor progression through non-enzymatic mechanisms remains to be elucidated. Third, beyond CAFs and pericytes, whether NNMT is dysregulated and functional in other stromal cells (e.g., endothelial cells, adipocytes) is largely unknown. Finally, known non-histone substrates downstream of NNMT are few. With the rapid expansion of multi-omics technologies and functional interrogation, it is anticipated that new NNMT-mediated post-translational modification targets will be uncovered, thereby broadening our understanding of its multifaceted roles in tumor biology.

In summary, NNMT integrates metabolic and epigenetic circuitry across tumor and stromal compartments, making it a compelling systems-level therapeutic node. Progress in selective chemistry (including bisubstrate designs and prodrugs), precision delivery to the TME, and companion biomarkers should accelerate translation. Strategically combining NNMT inhibition with ICB, targeted agents and metabolic blockade offers a credible path to durable benefit in NNMT-high cancers.

## Data Availability

No datasets were generated or analysed during the current study.

## Abbreviations

NNMT: Nicotinamide N-methyltransferase
SAM: S-adenosyl-L-methionine
NAM: Nicotinamide
MNAM: 1-methylnicotinamide
SAH: S-adenosyl-homocysteine
TME: tumor microenvironment
CAFs: Cancer-associated fibroblasts
H3K27me3: Histone H3 lysine 27 trimethylation
H3K4me3: Histone H3 lysine 4 trimethylation
TPCs: Tumor pericytes
H3K9me3: Histone H3 lysine 9 trimethylation
H3K18la: Histone H3 lysine 18 acetylation
ICB: Immune checkpoint blockade
